# Targeted therapy of non-small cell lung cancer: mechanisms and clinical trials

**DOI:** 10.3389/fonc.2024.1451230

**Published:** 2024-09-26

**Authors:** Le Yu, Ruoyi Yang, Zeng Long, Qingxiu Tao, Bin Liu

**Affiliations:** ^1^ Sichuan Cancer Hospital, University of Electronic Science and Technology of China, Chengdu, Sichuan, China; ^2^ School of Medical and Life Sciences, Chengdu University of Traditional Chinese Medicine, Chengdu, Sichuan, China

**Keywords:** NSCLC, target therapy, TKIs, monoclonal antibodies, antibody-drug conjugate

## Abstract

Lung cancer is a leading cause of cancer-related deaths globally, and traditional chemotherapy has limited efficacy in treating advanced non-small cell lung cancer (NSCLC). In recent years, the prognosis for patients with NSCLC has significantly improved due to the development of new treatment modalities, including targeted therapies. Targeted therapies utilize monoclonal antibodies (mAbs), antibody-drug conjugates (ADCs), or small molecule tyrosine kinase inhibitors (TKIs) directed against specific mutated genes such as EGFR and ALK. The development of these drugs has deepened our understanding of NSCLC and improved treatment outcomes for patients. This review aims to summarize the mechanisms and current status of targeted therapy for NSCLC, discuss strategies to overcome acquired resistance, and address current challenges in the field.

## 1 Introduction

Lung cancer is a leading cause of cancer deaths globally, accounting for 18% of cancer-related deaths (1). In China, it is the most common type of cancer, with approximately 630,500 deaths annually, representing 27% of all cancer deaths (2). Lung cancer is classified into multiple subtypes based on histology, with non-small cell lung cancer (NSCLC) comprising 85%, including adenocarcinoma (40%), squamous cell carcinoma (30%), large cell (undifferentiated) carcinoma (15%), and other rare types (3). Due to the lack of specific early screening methods, most NSCLC patients are diagnosed at advanced stages or with widespread metastasis, resulting in poor prognosis.

For many years, chemotherapy, radiation therapy, and surgical tumor resection have been the mainstays of NSCLC treatment, but advancements in chemotherapy have not significantly improved patient survival rates. Over the past decade, significant improvements in prognosis for lung cancer patients have been achieved due to research and application of new treatment modalities such as targeted therapy and immunotherapy. Targeted therapies, utilizing monoclonal antibodies (mAbs) or tyrosine kinase inhibitors (TKIs) against specific mutated genes such as EGFR and ALK, have greatly enhanced outcomes for lung cancer patients (4, 5). On this basis, the Antibody-Drug Conjugate (ADC) drugs have shown exciting results in the treatment of NSCLC (6). In addition, patients with advanced NSCLC, immunotherapy is an important treatment strategy, and its combination with other targeted therapies helps further improve patient prognosis (7). In this review, we aim to summarize the mechanisms and current status of targeted therapy for NSCLC, discuss strategies to overcome acquired resistance, and address current challenges in the field.

## 2 TKIs therapy

**Figure 1:**
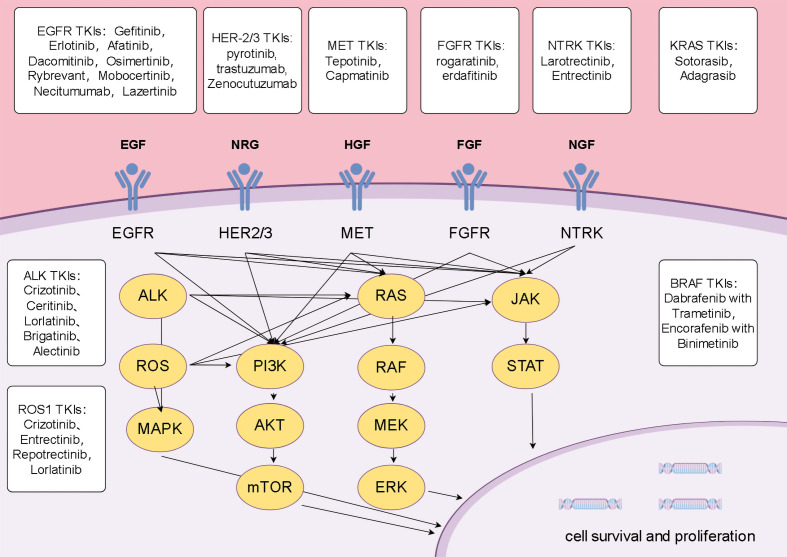
Mechanisms of lung cancer progression and potential therapeutic targets.

Tyrosine kinases (TKs) are crucial cell signaling enzymes that regulate pathways related to cell growth, differentiation, and apoptosis (8). Their oncogenic mutations or overexpression are markers of cell cycle dysregulation and play a role in the development and progression of various cancers, including NSCLC. TKIs selectively inhibit TK proteins, preventing tumor cell proliferation and growth, and play a significant role in targeted therapy for NSCLC (9). Due to this highly specific mechanism, TKIs often have minimal impact on normal cells, resulting in relatively fewer side effects, however, resistance remains a challenge in clinical cancer treatment (10). Some acquired resistance mechanisms have been identified, and further research into these targets and resistance mechanisms is essential for improving the prognosis of NSCLC (**Figure 1**).

### 2.1 EGFR

Epidermal growth factor receptor(EGFR)is a receptor tyrosine kinase, also known as ErbB1/human epidermal growth factor receptor 1 (HER1), belonging to the tyrosine kinase receptor ErbB family. This family also includes ErbB2/human epidermal growth factor receptor 2 (HER-2), ErbB3/human epidermal growth factor receptor 3 (HER-3), and ErbB4/human epidermal growth factor receptor 4 (HER4). Upon binding with growth factors, the domains of EGFR and other ErbB family members undergo phosphorylation, activating their cytoplasmic tyrosine kinase domains, thereby initiating intracellular signal transduction that affects cell proliferation and differentiation. Overexpression or mutation activation of EGFR is closely associated with the development and progression of many human malignancies (8, 11).

In lung cancer patients, EGFR mutation is one of the most common variants in NSCLC. In Asian populations, at least 50% of patients have EGFR gene mutations, with exon 19 deletions (Ex 19Del) and exon 21 single amino acid substitutions (L858R) being the most common. These mutations alter the receptor’s conformation, leading to dimerization and increased activity, while reducing affinity for ATP (12–14).This allows first-generation EGFR TKIs such as gefitinib, erlotinib, and afatinib to competitively and reversibly inhibit downstream signaling, significantly improving response and survival rates in untreated EGFR mutation-positive patients. Clinical results show erlotinib achieves a response rate (RR) of 72% and a median progression-free survival (PFS) of nearly 10 months in patients with EGFR mutations (Ex 19Del or L858R), while maintaining good safety profiles (15).

However, a considerable proportion of patients develop resistance to first-generation EGFR therapy, with 60% of resistant cases associated with the T790M gatekeeper mutation. This mutation increases receptor affinity for ATP, hindering competitive inhibition by first-generation EGFR TKIs (16, 17). To overcome this resistance mechanism, second-generation EGFR TKIs, such as afatinib and dacomitinib, were developed as irreversible inhibitors. They form irreversible covalent bonds with the C797 residue of EGFR, blocking ATP binding and controlling disease progression. Despite demonstrating significant PFS and overall survival (OS) benefits in first-line therapy, second-generation EGFR TKIs exhibit poor efficacy against T790M-mediated resistance to first-generation EGFR therapy due to their lower selectivity for the T790M EGFR mutation compared to wild-type (WT) EGFR. Additionally, their clinical use is associated with higher rates of adverse events compared to first-generation drugs (18).

Third-generation EGFR-TKIs, such as osimertinib, have shown significant efficacy in overcoming acquired resistance mediated by the T790M mutation from first- and second-generation EGFR-TKIs. These drugs also form irreversible covalent complexes with EGFR, not only overcoming enhanced ATP affinity conferred by the T790M mutation but also showing higher selectivity for L858R/T790M mutant EGFR, with 200 times the potency against WT EGFR, thus significantly improving treatment outcomes (19). For instance, results from the AURA3 clinical trial demonstrate that osimertinib is superior to platinum-based chemotherapy plus pemetrexed in terms of efficacy in T790M-positive advanced NSCLC patients who progressed during first-line EGFR-TKI therapy, achieving a 71% objective RR and a median PFS of 10.1 months in patients with advanced disease, including central nervous system metastases (20). The FLAURA trial further confirms osimertinib’s superiority over first-generation EGFRis in first-line treatment of EGFR mutation-positive advanced NSCLC, showing longer median PFS (18.9 months vs. 10.2 months), similar objective RR (80% vs. 76%), and lower rates of severe adverse events (34% vs. 45%) (21). However, even third-generation EGFR-TKIs cannot avoid the development of resistance. Resistance mechanisms to third-generation EGFR-TKIs in advanced NSCLC are highly complex and vary between patients, highlighting the critical importance of next-generation sequencing (NGS) testing for evaluating resistance mechanisms and identifying subsequent treatment targets. Currently, novel fourth-generation EGFR-TKIs are under preclinical investigation, aiming to overcome existing EGFR-mediated acquired resistance and provide new perspectives for subsequent treatment of EGFR mutation-positive patients (22, 23).

### 2.2 ALK

The ALK gene encodes a tyrosine kinase receptor belonging to the insulin receptor superfamily. Its activation leads to receptor dimerization and autophosphorylation, subsequently activating downstream signaling pathways crucial for cell proliferation, migration, and differentiation. In malignant tumors, most ALK gene mutations occur in the form of translocations with another partner gene, leading to aberrant activation of ALK and its downstream signaling pathways, thereby promoting tumor progression (24, 25).In NSCLC, ALK fusion genes are present in approximately 3%-5% of patients, more commonly observed in younger individuals (26). Among these, the EML4-ALK fusion, involving the echinoderm microtubule-associated protein-like 4 (EML4) gene, is the most frequent, found in about 85% of all ALK fusion cases. Additionally, ALK gene rearrangements with partner genes such as KIF5B, TFG, TPR, BCL11A, though the choice of ALK inhibitors currently does not depend on the specific fusion type (27–30).

Crizotinib is a first-generation multi-targeted ALK inhibitor that also exhibits activity against MET and ROS1 (31). Clinical studies have shown a RR of 65% with crizotinib, and a median PFS of 7.7 months, which is superior to standard chemotherapy, though it has not significantly improved OS compared to chemotherapy (32). Second-generation ALK inhibitors include ceritinib and alectinib, which have higher affinity for ALK and better penetration through the blood-brain barrier. Alectinib has demonstrated higher PFS rates compared to crizotinib, with a 12-month event-free survival rate of 68.4% (33). Similar to alectinib, the brigatinib group shows a higher PFS compared to the crizotinib group (34)..

Most patients inevitably develop treatment resistance after receiving second-generation ALK TKI therapy. The G1202R mutation is the most common acquired resistance mutation among patients treated with second-generation ALK inhibitors. Its occurrence rates in patients treated with ceritinib, alectinib, and brigatinib are 21%, 29%, and 43%, respectively (35, 36). Lorlatinib, a reversible third-generation ALK and ROS1 inhibitor, overcomes multiple ALK resistance mutations including G1202R, and exhibits good blood-brain barrier penetration (37). Clinical studies indicate that in ALK-positive patients who have previously received at least one ALK TKI, the objective RR of lorlatinib is 47.0%. Among patients who have previously received two or more ALK TKIs, the objective RR is 38.7% (38). CROWN trial assessed the efficacy of lorlatinib in treatment-naive, ALK-positive NSCLC patients. The results showed that the 3-year PFS rate was 64% in the lorlatinib group compared to 19% in the crizotinib group. Compared to crizotinib, lorlatinib demonstrated improvements in PFS, objective RR, intracranial objective RR, intracranial progression time, and duration of response, indicating durable benefits with lorlatinib (39).Apart from further refining treatment targets, immunotherapy may represent a new strategy for the treatment of ALK-positive NSCLC after development of resistance. The complex interaction between ALK rearrangements and immune cells suggests that high PD-L1 expression could serve as a poor prognostic biomarker in ALK-positive NSCLC, indicating that immune checkpoint inhibitors combined with targeted therapy may potentially improve patient outcomes (40).

### 2.3 ROS1

ROS1 is an oncogene located on chromosome 6Q22.1 that encodes a member of the insulin receptor subfamily. ROS1 fusion genes have been identified in various types of tumors, with the most prevalent fusion being CD74-ROS1, found in 44% of cases, followed by EZRs-ROS1, SDC4-ROS1, and SLC34A2 (41–43). The ROS1 fusion protein activates downstream signaling pathways to promote cell proliferation, activation, and cell cycle progression, accelerating the development and progression of NSCLC. ROS1 rearrangements are detected in 1%-2% of NSCLC patients, and 36% of ROS1-positive NSCLC patients harbor concurrent oncogenic mutations such as EGFR or KRAS mutations, MET amplification, or ALK translocations (44). The central nervous system is a common site of metastasis in ROS1 fusion-positive NSCLC patients, with up to 36% diagnosed with brain metastases, and many others potentially developing intracranial metastases subsequently. Therefore, treatment strategies for ROS1-positive NSCLC patients should encompass the central nervous system, and ROS1 testing is recommended for all NSCLC patients with brain metastases (45).

ROS1 belongs to the insulin receptor family. While not all ALK TKIs exhibit dual inhibitory activity against both ALK and ROS1, several TKI targeting multiple ALK targets have shown efficacy in treating ROS1. Due to its unique structure, crizotinib inhibits ROS1 with five times the potency compared to ALK, showing significant efficacy in ROS1-mutant patients. Results from the PROFILE 1001 study demonstrate an objective RR of 72% with crizotinib in advanced ROS1-rearranged NSCLC patients, with a median duration of response of 24.7 months. Consistent objective RRs were observed across different subgroups, with a median PFS of 19.3 months and a median OS of 51.4 months (46). The EUCROSS study evaluated crizotinib in ROS1-positive NSCLC, reporting an objective RR of 70% and a median PFS of 20.0 months. These studies underscore the therapeutic efficacy of crizotinib while indicating a poorer prognosis for patients with brain metastases, likely due to its limited ability to penetrate the blood-brain barrier. In contrast, entrectinib effectively crosses the blood-brain barrier, offering an advantage in treating patients with central nervous system metastases (47). Multiple studies confirm entrectinib’s role in ROS1-mutant NSCLC, demonstrating an objective RR of 77% and a comprehensive RR of 67.1%, with 12-month PFS and OS rates of 55% and 81%, respectively. In patients with central nervous system metastases, entrectinib achieves an intracranial objective RR of 79.2% and a median intracranial PFS of 12.0 months (48).

Lorlatinib, a third-generation TKI targeting the kinase domains of ALK and ROS1, has shown efficacy in NSCLC patients resistant to first and second-generation ALK inhibitors. Studies in advanced ROS1-positive NSCLC demonstrate an objective RR of 62% in TKI-naïve patients and 35% in patients previously treated with crizotinib. Lorlatinib achieves a 64% intracranial RR in TKI-naïve patients and 50% in crizotinib-resistant patients (49). However, the PFROST study indicates limited efficacy of lorlatinib in treating secondary ROS1 resistance mutations induced by crizotinib (50). Mechanisms of acquired resistance to ROS1 inhibitors are still under investigation, and several potential therapeutic agents have been developed to further enhance treatment outcomes for ROS1-mutant patients.

### 2.4 BRAF

BRAF is a serine/threonine protein kinase belonging to the RAF kinase family. While all RAF proteins can phosphorylate MEK (MEK1 and MEK2), BRAF exhibits the strongest activation capability. Upon activation by RAS, BRAF further activates MEK to phosphorylate ERK (ERK1 and ERK2) in the cytoplasm, which subsequently translocates to the nucleus. In the nucleus, ERK1/2 phosphorylates and activates various transcription factors, thereby participating in the regulation of apoptosis, proliferation, migration, and enhancing the expression of genes involved in many oncogenic processes (51–54).BRAF mutations occur in 3-5% of NSCLCs, predominantly in adenocarcinoma histology. Based on their dimerization status, kinase activity, and RAS dependency, BRAF mutations are classified into three types. Type 1 mutations, characterized by a monomeric state and high BRAF kinase activity independent of RAS, include the most common V600E point mutation found in 90% of BRAF-mutant tumors on exon 15. Compared to type 1 alterations, type 2 and type 3 mutations are associated with a higher risk of brain metastases at diagnosis (55–58).

The role and clinical significance of BRAF mutations in solid tumors have been extensively studied, leading to the development of various BRAF TKIs and therapeutic strategies targeting BRAF mutations. Following positive results in metastatic melanoma patients with BRAF V600E mutations, anti-BRAF therapies have been extended to other tumor types harboring the same mutation. The EURAF cohort compared dabrafenib monotherapy with dabrafenib in combination with trametinib, showing an overall RR of 53% and a disease control rate of 85% for BRAF-targeted therapy. Median PFS with BRAF-targeted therapy was 5.0 months, and OS was 10.8 months (59).

Results from phase II clinical trials of dabrafenib-trametinib therapy in previously treated metastatic BRAF V600E mutation NSCLC patients showed an overall RR of 63.2%. In treatment-naïve metastatic BRAF V600E mutation NSCLC, first-line dabrafenib combined with trametinib achieved an overall RR of 64% (60, 61). Based on these findings, dabrafenib-trametinib combination therapy has been FDA-approved as first-line treatment for BRAF V600 mutant lung cancer (62). Apart from dabrafenib-trametinib, FDA approvals also include vemurafenib/cobimetinib and encorafenib/binimetinib for advanced or metastatic NSCLC with BRAF (V600E/K) mutations.

### 2.5 MET

The MET gene, located on chromosome 7q21-q31, encodes the protein tyrosine kinase MET, a transmembrane receptor in the RTK family. MET is activated by HGF, regulating cell growth and development by promoting mitosis, movement, and invasion. Pathological MET activation drives tumorigenesis in various cancers by enhancing proliferation, invasive growth, and angiogenesis (63, 64). Oncogenic MET alterations include mutations, amplifications, overexpression, chromosomal rearrangements, and fusions, disrupting the HGF/MET axis and contributing to cancers like NSCLC. METex14 skipping mutations, found in 3%-4% of NSCLC patients, are linked to poor prognosis and responsiveness to MET-targeted therapy, serving as a predictive biomarker for MET TKI sensitivity (65–67). The PROFILE 1001 study showed that crizotinib monotherapy was effective for advanced NSCLC patients with METex14 mutations (38% treatment-naive): objective response rate was 32%, median duration of response was 9.1 months, and median progression-free survival was 7.3 months. Crizotinib is approved for previously platinum-treated, metastatic NSCLC patients with METex14 mutations.Tepotinib is a selective MET inhibitor that suppresses tumor growth by disrupting MET signaling pathways. Results from the VISION study shows that in NSCLC patients with MET exon 14 skipping mutations, the ORR was 56%. The median PFS was 8.9 months. There was no statistically significant difference in outcomes between treatment-naïve and previously treated patients (68).

In addition to influencing tumor progression through various mechanisms, MET amplification is recognized as a distinct mechanism of acquired resistance to EGFR TKIs (EGFR-TKIs) in advanced EGFR-mutant NSCLC patients. MET amplification is detected in 5% to 22% of NSCLC patients resistant to first-generation EGFR TKIs, representing the second most common acquired resistance mechanism after the EGFR T790M mutation (69).Combination therapy with MET TKIs and EGFR-TKIs has shown significant clinical benefits for this patient subset, demonstrating preliminary clinical activity with a Phase Ib/II ORR of 27%. Increased activity has been observed particularly in patients with high MET amplification levels; in patients with MET gene copy number ≥6, the Phase II ORR was 47% (70).Thus, MET TKIs have become a potential effective option for patients who have developed resistance to EGFR-TKIs. Several new MET TKIs are currently under development and in clinical trials, aiming to provide new opportunities for patients with MET mutations (71).

### 2.6 KRAS

The Kirsten rat sarcoma viral oncogene homolog (KRAS), along with its homologs NRAS and HRAS, belongs to the RAS GTPase protein family, situated on the inner surface of the cell membrane. It dynamically regulates between an inactive GDP-bound state (KRAS-GDP) and an active GTP-bound state (KRAS-GTP), acting as a molecular switch in signaling cascades that govern cell proliferation, survival, and differentiation (72).KRAS gene mutations lead to an increase in KRAS-GTP state, thereby triggering downstream oncogenic pathways. KRAS mutations are among the most common genomic alterations in solid tumors, accounting for 85% of observed RAS mutations in human cancers. Among various KRAS mutation variants, KRAS G12C is the most prevalent, and occurs in 13% of NSCLC cases (73). KRAS-driven cancers depend on sustained activation and signaling of KRAS, making it an ideal therapeutic target. However, due to the lack of deep binding pockets for specific small molecule inhibitors, it has historically been considered an “undruggable” target (74).

In clinical practice, first-line platinum-based chemotherapy ± immunotherapy is the recommended choice for KRAS-mutant patients. Attempts targeting KRAS have not been particularly successful. Research results of sotorasib in patients with advanced NSCLC harboring KRAS p.G12C mutation who have previously received standard therapy show an objective RR of 37.1%. The median duration of response is 11.1 months. The median PFS is 6.8 months, and the median OS is 12.5 months (75). In May 2021, the FDA accelerated the approval of sotorasib for the treatment of locally advanced or metastatic NSCLC in adults with KRAS (G12C) mutation who have received at least one prior FDA-approved systemic therapy. Further clinical trials are ongoing to bring new strategies for NSCLC treatment (71).

Based on clinical trial results, the FDA has approved various TKI drugs for the treatment of NSCLC (**Table 1**). With the deepening of fundamental research and the continuous improvement of genetic testing methods, understanding of NSCLC has advanced further. More potential therapeutic targets for NSCLC, such as PIK3CA and HER-2, are being explored (76–78). These ongoing studies and the development of next-generation inhibitors aim to enhance efficacy, reduce side effects, and address current drug resistance, potentially leading to better outcomes for NSCLC patients in the future (79). Besides new drug development, novel treatment strategies, such as combining targeted therapy with chemotherapy or immunotherapy, are being developed to further improve patient prognosis (80). With the advancement of new-generation testing technologies like NGS, we will be able to obtain more comprehensive and convenient molecular profiles of NSCLC tumors, enabling personalized drug selection and the formulation of optimal treatment strategies.

**Table 1:** FDA-approved TKI drugs for NSCLC treatment.

Target	Common mutation sites/fusion type	FDA-approved drugs
EGFR	EGFR L858R,EGFR Ex19del	Gefitinib,Erlotinib,Afatinib,Dacomitinib,Osimertinib,Rybrevant,Mobocertinib,Necitumumab,Lazertinib with Amivantamab-vmjw,
ALK	EML4-ALK fusion	Crizotinib,Ceritinib,Lorlatinib,Brigatinib,Alectinib,
ROS1	CD74-ROS1	Crizotinib,Entrectinib,Repotrectinib,Lorlatinib
BRAF	BRAF V600E	Dabrafenib with Trametinib,Encorafenib with Binimetinib
KRAS	KRAS G12c	Sotorasib,Adagrasib
NTRK	NTRK 1/2/3	Larotrectinib,Entrectinib
MET	METex14	Tepotinib,Capmatinib,
RET	RET-KIF5B	Selpercatinib,Pralsetinib

## 3 Monoclonal antibody therapy

Conventional chemotherapy and/or radiotherapy have shown limited efficacy in treating NSCLC patients, partly due to the high doses required for tumor eradication, which often lead to irreversible damage to normal tissues (81). As a more precise treatment, monoclonal antibody therapy not only enhances therapeutic efficacy for patients but also reduces side effects, thereby improving patient tolerance and compliance with treatment. Approved biologics for treating NSCLC include cetuximab, bevacizumab, nivolumab, and pembrolizumab. Among them, nivolumab, pembrolizumab, and immunotherapy are highly correlated.

### 3.1 EGFR monoclonal antibodies

Cetuximab is a recombinant chimeric human/mouse IgG1 monoclonal antibody that binds to the EGFR and competitively inhibits the binding of epidermal growth factor (EGF) and other ligands, thereby blocking ligand-induced EGFR phosphorylation and downstream signaling pathways (82). In advanced lung cancer patients, the role of cetuximab in combination with conventional chemotherapy remains unclear. Clinical study results of cetuximab combined with paclitaxel/carboplatin as first-line therapy for advanced NSCLC show: median PFS was 4.40 months in the cetuximab group and 4.24 months in the TC (Taxane/Carboplatin) group. Median OS was 9.69 months in the cetuximab group and 8.38 months in the TC group. ORR was 25.7% in the cetuximab group and 17.2% in the TC group. No significant benefit was observed in advanced NSCLC patients (83).

### 3.2 VEGF monoclonal antibodies

VEGF monoclonal antibodies promote tumor growth by enhancing endothelial cell proliferation and survival, increasing endothelial cell migration and invasion, increasing vascular permeability, and enhancing chemotaxis and homing of bone marrow-derived precursor cells (84). Bevacizumab is a humanized monoclonal antibody against VEGF that reduces tumor expansion by controlling abnormal vascular growth around tumors. It was the first anti-tumor angiogenesis drug approved for first-line treatment of metastatic colorectal cancer by the FDA in 2004. The expression of HER-1/EGFR and VEGF molecules in NSCLC is associated with poor prognosis. The AVAil trial evaluated cisplatin/gemcitabine (CG) plus bevacizumab in advanced non-squamous NSCLC, with a high-dose PFS of 6.5 months, a low-dose PFS of 6.7 months, and a control group of 6.1 months, with ORRs of 20.1%, 34.1%, and 30.4%, respectively, for placebo, low-dose bevacizumab, and high-dose bevacizumab plus CG, suggesting that bevacizumab (7.5 or 15 mg/kg) in combination with CG significantly improves PFS and objective RR (85). The role of bevacizumab combined with chemotherapy (docetaxel or pemetrexed) or erlotinib in refractory non-squamous NSCLC suggests a one-year survival rate of 57.4% for bevacizumab-erlotinib, 53.8% for bevacizumab-chemotherapy, and 33.1% for chemotherapy alone. The results of PFS and OS favor bevacizumab combined with chemotherapy or erlotinib over monotherapy (86).

## 4 Antibody-drug conjugate

**Figure 2:**
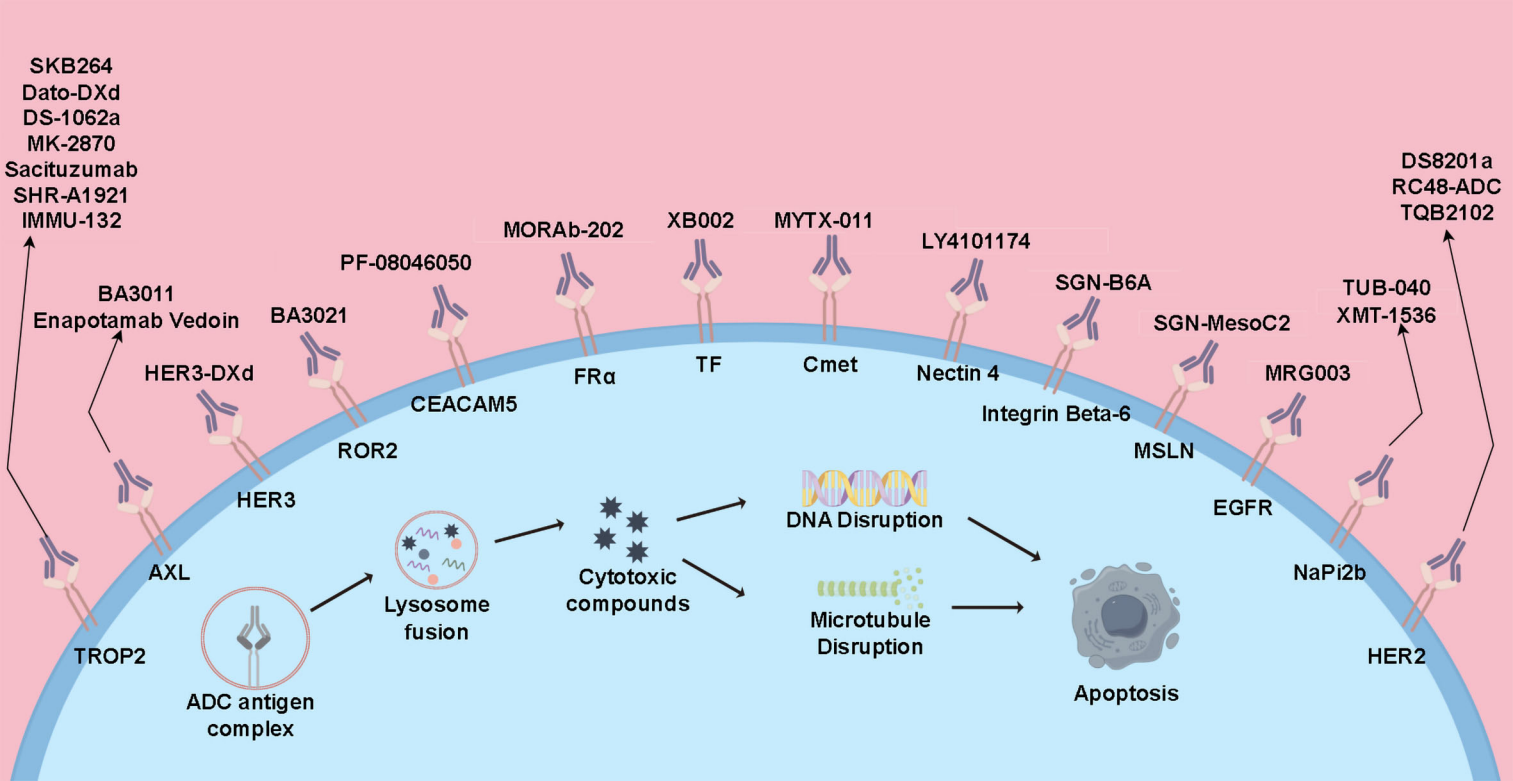
Mechanisms of action of ADCs and current therapeutic drugs.

ADC drugs are a novel class of anti-tumor medications that link small molecule cytotoxic drugs with monoclonal antibodies through linkers. These drugs can specifically target tumor cells, combining the potent cytotoxic effects of traditional small molecule chemotherapy with the tumor-targeting properties of antibody drugs (6, 87). Currently, several ADC drugs are under research in the field of lung cancer and have shown significant clinical efficacy (**Figure 2**).

### 4.1 HER-2 ADC therapy

Trastuzumab deruxtecan (T-DXd; DS-8201) consists of a humanized anti-HER-2 monoclonal antibody (trastuzumab) linked to a topoisomerase I inhibitor. Previous clinical trials have validated its efficacy in HER-2-positive breast and gastric cancers, leading to its approval for metastatic HER-2-positive breast cancer and gastric cancer (88, 89). DESTINY-Lung01 evaluated T-DXd in refractory HER-2-overexpressing or HER-2-mutant NSCLC, showing a median PFS of 8.2 months, median OS of 18.6 months, median DOR of 10.6 months, and DCR of 92.3%, demonstrating its efficacy in treating HER-2-positive NSCLC patients (90). DESTINY-Lung02 studied the efficacy and safety of trastuzumab deruxtecan (DS-8201) in previously treated HER-2-mutant advanced NSCLC. In patients receiving 5.4 mg/kg dose of trastuzumab deruxtecan, the ORR was 49%, with median PFS of 9.9 months. In the 6.4 mg/kg dose group, the ORR was 56.0%, and median PFS reached 15.4 months (91). Based on these trials, T-DXd has been recommended for treating previously treated HER-2-mutant advanced NSCLC patients.

T-DM1 is an ADC drug that consists of an anti-HER-2 antibody (trastuzumab) linked to the microtubule inhibitor emtansine (DM1) via a non-cleavable thioether linker. In single-agent treatment, it achieved an ORR of 6.7% in recurrent HER-2-positive NSCLC. The median follow-up time was 9.2 months, with median PFS and median OS of 2.0 months and 10.9 months, respectively. Due to limited efficacy, the study was terminated early (92). This treatment approach did not achieve satisfactory results in other studies involving HER-2-positive NSCLC, warranting further investigation into its efficacy (93, 94).

### 4.2 HER-3 ADC therapy

HER-3-DXd is an antibody-drug conjugate composed of a HER-3 antibody linked to a topoisomerase I inhibitor. HER-3 receptor tyrosine kinase protein is expressed in most EGFR-mutant lung cancers, although it is not a known mechanism of resistance to EGFR inhibitors. The HETHENA-Lung01 trial evaluated the efficacy and safety of patritumab deruxtecan in locally advanced or metastatic NSCLC patients with EGFR mutations who progressed on EGFR-TKI therapy. It reported an ORR of 29.8%, median PFS of 5.5 months, and median OS of 11.9 months. The study observed efficacy across a broad range of tumor HER-3 membrane expression levels and different mechanisms of EGFR TKI resistance (95).

### 4.3 TROP-2 ADC therapy

Datopotamab deruxtecan (Dato-DXd) is an ADC drug targeting the transmembrane protein TROP-2, consisting of a humanized anti-TROP-2 IgG1 monoclonal antibody conjugated with a topoisomerase I inhibitor. Currently, Dato-DXd is undergoing multiple studies in the field of lung cancer. The TROPION-Lung01 trial aimed to evaluate the efficacy and safety of Dato-DXd monotherapy compared to docetaxel in patients with advanced NSCLC who had received at least one prior treatment, with or without driver gene mutations. The Dato-DXd group demonstrated a significant PFS (PFS) benefit compared to the docetaxel group (4.4 months vs. 3.7 months). It is noteworthy that in the non-squamous subgroup, patients showed better PFS with Dato-DXd compared to docetaxel, with PFS of 5.6 months and 3.7 months, respectively (96).

TROPION-Lung02 evaluated the safety and efficacy of Dato-DXd (at 4 or 6 mg/kg) plus pembrolizumab ± platinum-based chemotherapy in patients with advanced NSCLC in the first-line or previously treated setting. The results showed an ORR of 38% in the doublet group and 49% in the triplet group. In the first-line treatment population, the ORR was 50% in the doublet group and 57% in the triplet group (97).

Sacituzumab govitecan is another ADC targeting TROP-2. The EVOKE-02 trial evaluated sacituzumab govitecan in combination with pembrolizumab as first-line treatment for patients with advanced or metastatic NSCLC negative for driver gene mutations. The results showed an ORR of 55.5% and a disease control rate (DCR) of 82% in the patient population. In the subgroup of patients with PD-L1 tumor proportion score (TPS) ≥50%, the ORR was notably higher at 69%, with a DCR of 86%, demonstrating promising early efficacy and manageable safety (98).

ADCs have received extensive attention in the treatment of NSCLC and have shown promising results in clinical trials. However, the complex combination of antibody and drug brings not only strong efficacy but also a high incidence of toxic side effects. A large-scale meta-analysis of existing ADC clinical trials indicates that the overall incidence of any-grade adverse events is 100.0%, and the incidence of ≥3 grade adverse events is 6.2%, limiting their further clinical use (99). The toxicity of ADCs mainly arises from two sources: the toxicity of the antibody molecule and the toxicity of the cytotoxic compounds. The antibody-induced toxicity mainly stems from damage to normal tissues expressing the target antigen, varying greatly based on target and patient expression. The cytotoxic compounds toxicity is primarily due to the toxin molecules’ release into the bloodstream, causing damage to normal cells, or non-specific internalization of ADCs by normal cells, often resulting in adverse effects similar to those of chemotherapeutic agents, such as hematologic toxicity, alopecia, and gastrointestinal reactions (6, 87).

In the DESTINY-Lung02 study, NSCLC patients were treated with Trastuzumab Deruxtecan at doses of 5.4 mg/kg and 6.4 mg/kg every 3 weeks. The results showed that 96% of patients in the low-dose group reported any-grade adverse events. Among them, 38.6% in the low-dose group experienced drug-related ≥3 grade AEs (91).In the TROPION-Lung01 study, NSCLC patients were treated with docetaxel or datopotamab deruxtecan. Results showed 25% and 41% of patients had grade 3 or higher TRAEs in the datopotamab deruxtecan and docetaxel groups, respectively. The Dato-DXd group had a higher incidence of grade 3 or higher drug-related ILD (3% vs. 1%) (100).

The high clinical efficacy of ADCs, coupled with their high incidence of adverse events, limits their further clinical application. Research on ADCs aims to develop safer and more effective drugs through mechanism exploration and to identify better dosing strategies through clinical trials to benefit patients. In new drug development, peptide-drug conjugates (PDCs) are a potential option, where antibody-binding regions are masked by unique peptides. Protease activity in the tumor microenvironment can cut these masking peptides, allowing PDCs to bind to target antigens and reduce toxicity (101, 102). Additionally, developing bispecific antibodies targeting two tumor-associated antigens, which strongly bind only to cells co-expressing both antigens, could further enhance drug selectivity. Improving the cytotoxicity of the toxin and the stability of the linker can also enhance drug efficacy (103). Clinical trials are exploring optimal drug dosing and combination strategies, such as ADCs with chemotherapy or immunotherapy, to benefit patients. Several drugs targeting NSCLC are currently under development and undergoing related clinical trials, with the hope that these studies will provide new treatment options for NSCLC patients (**Table 2**).

**Table 2:** Clinical trials of ADC therapies for NSCLC currently underway.

ADC name	target	NCT number
SKB264	TROP-2	NCT05351788,NCT06448312,NCT05870319,NCT05816252,NCT04152499,NCT06305754,NCT06074588,NCT06170788,NCT04152499
DS-1062a	TROP-2	NCT04656652,NCT03401385,NCT05460273,NCT06417814,NCT03944772,NCT04526691,NCT04612751
Dato-DXd	TROP-2	NCT06417814,NCT05460273,NCT05215340,NCT06357533,NCT04612751,NCT04526691,NCT05555732,NCT05061550,NCT06350097,NCT05687266,
Sacituzumab	TROP-2	NCT06312137,NCT05609968,NCT06422143,NCT05089734,NCT06055465,NCT06170788,NCT06431633,NCT05186974,NCT06074588,NCT06305754
SHR-A1921	TROP-2	NCT06434103,NCT06465238,NCT06480136
sacituzumab govitecan	TROP-2	NCT05609968,NCT06055465,NCT05089734,NCT06431633,NCT05186974,NCT06401824,
MK-2870	TROP-2	NCT06049212
DS8201a	HER-2	NCT04042701,NCT04686305,NCT03334617,NCT05246514,NCT05048797,NCT06250777
RC48-ADC	HER-2	NCT05745740,NCT06003231,NCT05847764,NCT06185400
TQB2102	HER-2	NCT06496490
HER-3-DXd	HER-3	NCT06222489,NCT04676477,NCT04619004,NCT05865990,NCT05338970,
TUB-040	NaPi2b	NCT06303505
XMT-1536	NaPi2b	NCT03319628
BA3011	AXL	NCT04681131
Enapotamab Vedotin	AXL	NCT02988817
BA3021	ROR2	NCT03504488,NCT04681131
MYTX-011	c-MET	NCT05652868
REGN5093-M114	c-MET	NCT04982224,NCT04077099
MRG003	EGFR	NCT05351788
SGN-B6A	Integrin Beta-6	NCT06012435,NCT06549816,NCT04389632
PF-08046050	CEACAM5	NCT06131840
SGN-MesoC2	MSLN	NCT06466187
MORAb-202	FRα	NCT05577715,NCT04300556
LY4101174	Nectin-4	NCT06238479
XB002	TF	NCT04925284
M1231	MUC1/EGFR	NCT04695847
BL-B01D1	EGFR/HER-3	NCT05983432, NCT05880706, NCT06498986, NCT06382129, NCT05956587, NCT06475300, NCT06382116
amivantamab	EGFR/c-MET	NCT05801029, NCT05845671, NCT06083857, NCT05601973, NCT04965090, NCT04599712, NCT02609776,NCT06247826, NCT04538664, NCT06120140,
PF-06647020	PTK7	NCT04189614
CX-2029	CD71	NCT03543813

Despite years of progress in basic research and clinical trials, lung cancer remains one of the deadliest diseases globally, attracting extensive research attention. we summarized several current major targets used in treatment and clinical trials, and provides an initial overview of the resistance mechanisms associated with these targets and current resistance-related treatment approaches. In addition to the main targets mentioned in this article, numerous other targets are under investigation and clinical trials. New treatment modalities, including immunotherapy combinations and multi-targeted TKIs, are expected to further optimize targeted treatment outcomes.
